# Supplementary material for: Genetic determinants of gene amplifications alter frequency and evolutionary trajectory of antibiotic resistance in Staphylococcus aureus
Source: bioRxiv. 2025 Apr 11:2025.04.10.647645. Preprint. [Version 1] doi: 10.1101/2025.04.10.647645 (PMC12190916; doi:10.1101/2025.04.10.647645)
Supplement: Supplement 1 [file media-1.pdf]

SUPPLEMENTARY FIGURES

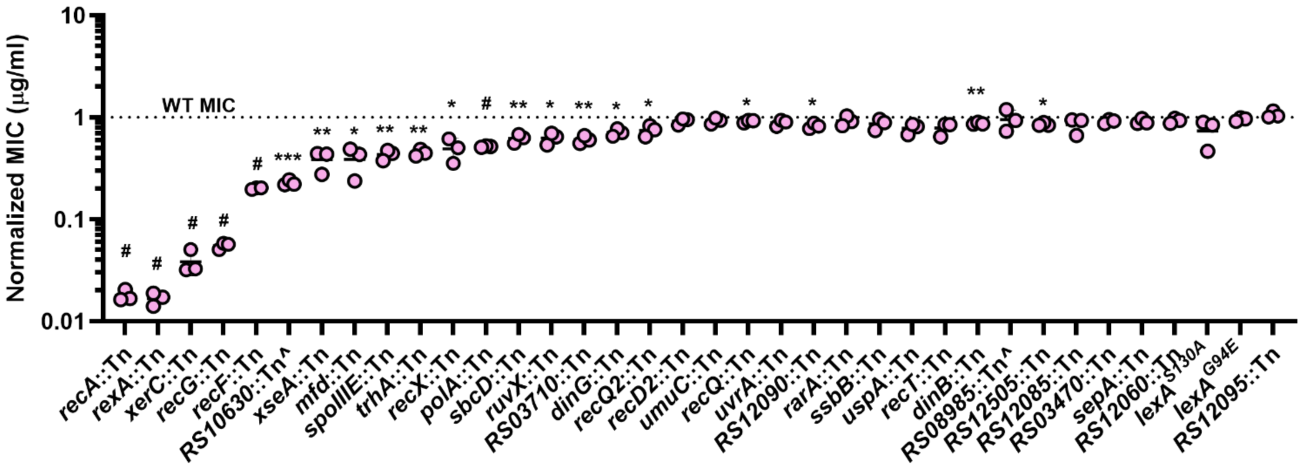

Supp. Fig. 1. Selected mutants show a wide range of DLX sensitivities. DLX MICs of WT and the indicated mutants were measured, and the mutant MICs were normalized to the WT MIC. Data shown are the mean ± standard deviation for three biological replicates. Significance is shown for comparison to a value of 1, as tested by one-sample *t*-tests (* $p < 0.05$, ** $p < 0.01$, *** $p < 0.001$, # $p < 0.0001$).

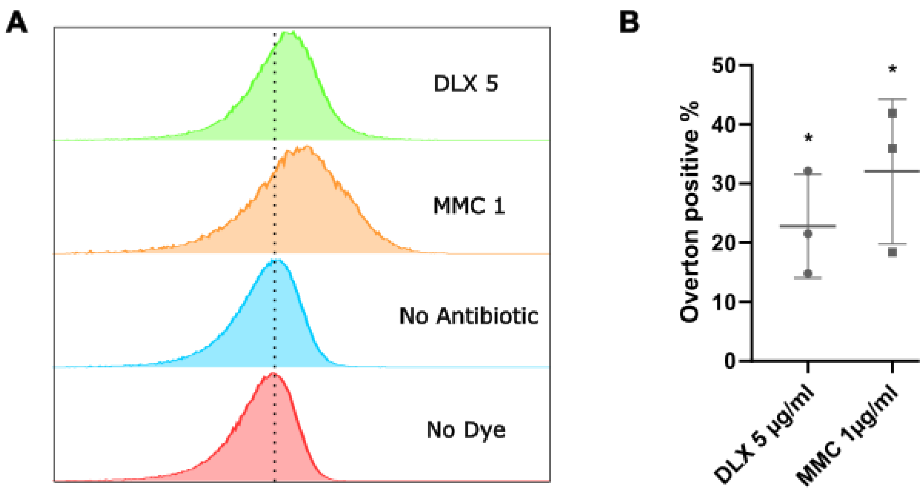

Supp. Fig. 2. DLX causes DNA damage. (A) Representative fluorescence from TUNEL staining of WT cells treated with no antibiotic, 1 µg/mL MMC, or 5 µg/mL DLX, or a no dye control. **(B)** The Overton positive percentage (representing the percentage of the population that has increased fluorescence compared to the control no antibiotic population) for the DLX and MMC treated samples. Significance is shown for comparison to a value of 0, as tested by one-sample *t*-tests (* $p < 0.05$).

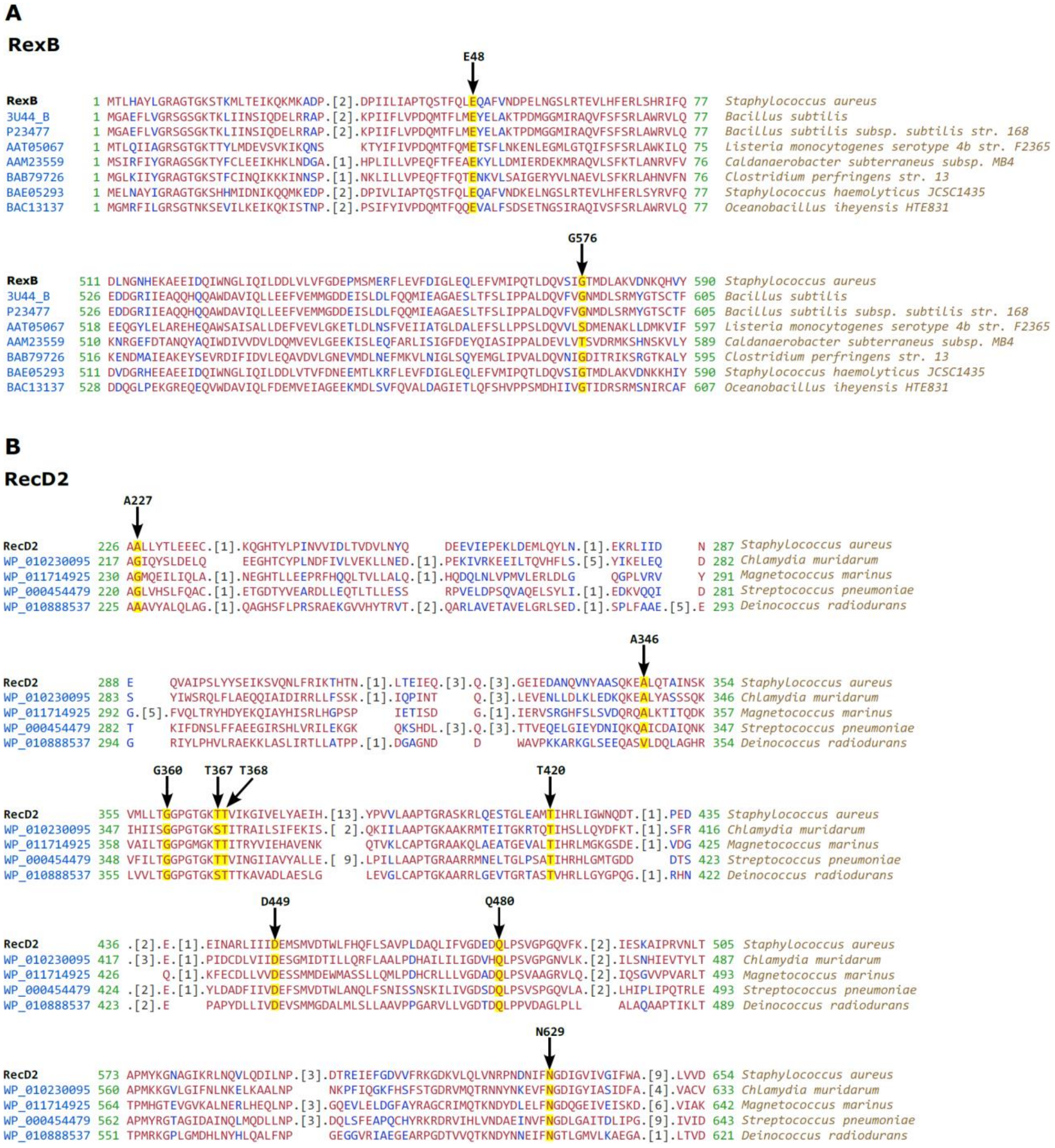

Supp. Fig. 3. Mutations seen in RexB and RecD2 are in relatively conserved residues. (A) and (B) Multiple sequence alignments of **(A)** RexB and **(B)** RecD2 from the Conserved Domain Database[1,2], where residues in red are highly conserved, and those in blue are not. Arrows denote the position of mutations, which are also highlighted, seen in the *recA*::Tn evolved populations.

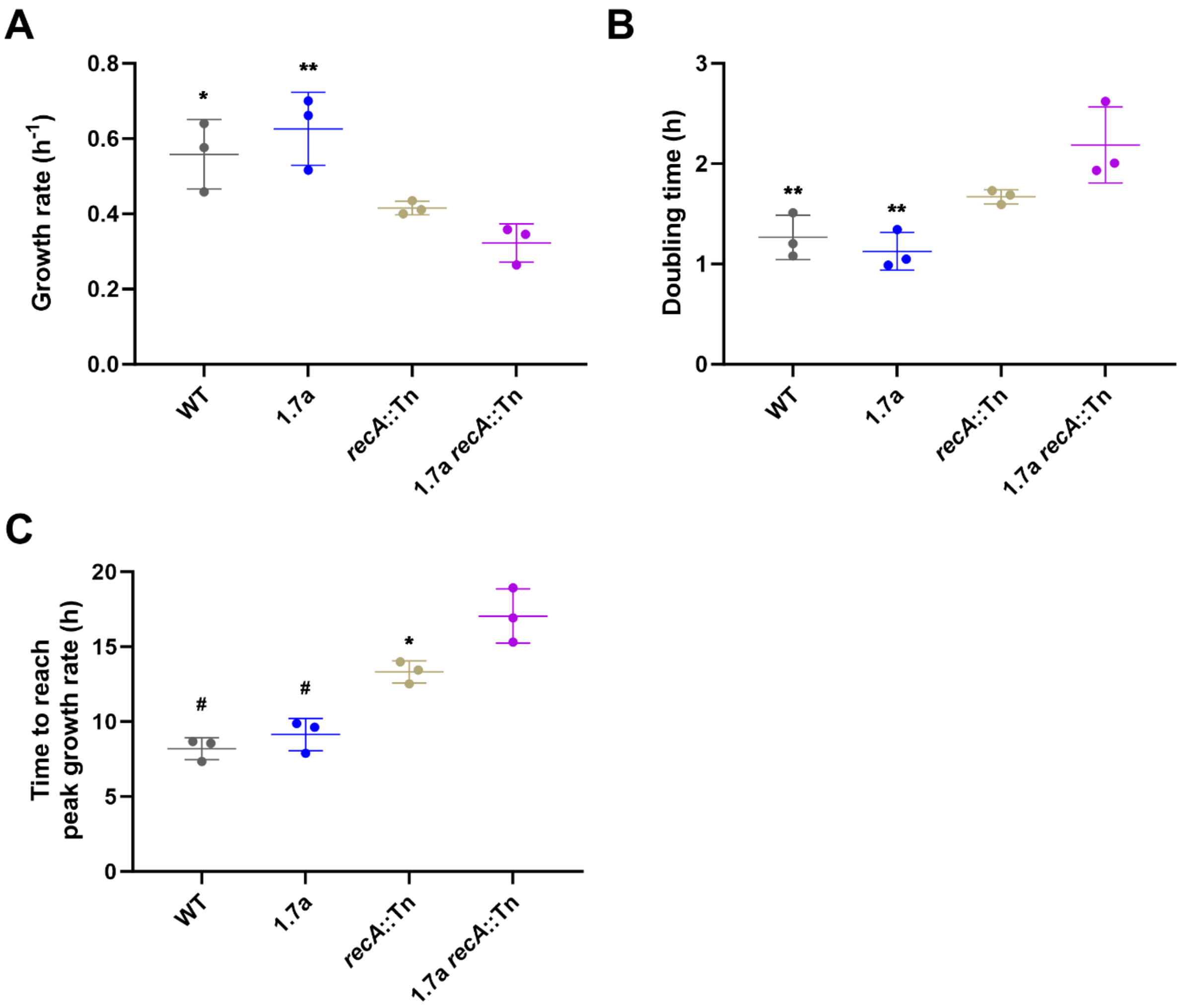

Supp. Fig. 4. Amplifications of *sdrM* have a fitness cost. (A) Growth rates, **(B)** doubling times, and **(C)** time to reach the peak growth rate are shown for the WT, and 1.7a, and *recA*::Tn mutants in both backgrounds. These parameters were obtained from growth curves shown in **Figure 3D** using Growthcurver[3] for fitting and analysis. Data shown are the means ± standard deviation for three biological replicates. Significance is shown for comparison to the 1.7a *recA*::Tn strain, as tested by a one-way ANOVA with Dunnett's test for multiple comparisons (* $p < 0.05$, ** $p < 0.01$, # $p < 0.0001$).

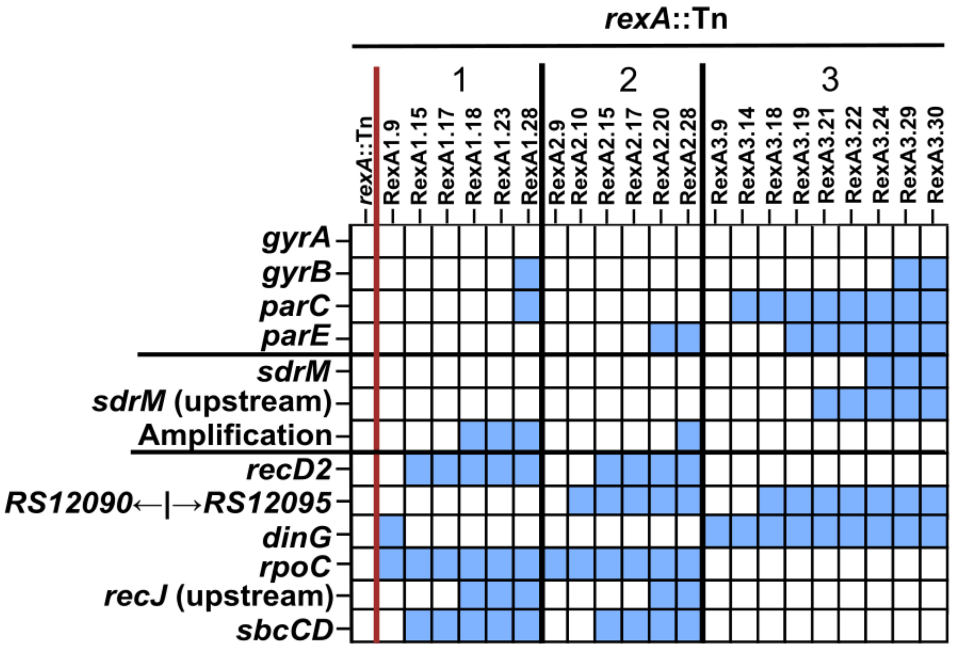

Supp. Fig. 5. The *rexA*::Tn mutant evolves DLX resistance initially via DNA repair-associated mutations, and later via mutations in the canonical targets, *sdrM*, mutations increasing *sdrM* expression, and *sdrM* amplifications. Common mutations seen in three independently evolved populations of the *rexA*::Tn mutant. Presence of mutations in the corresponding genes is indicated by a blue square. For each population, passages are shown from left to right in chronological order.

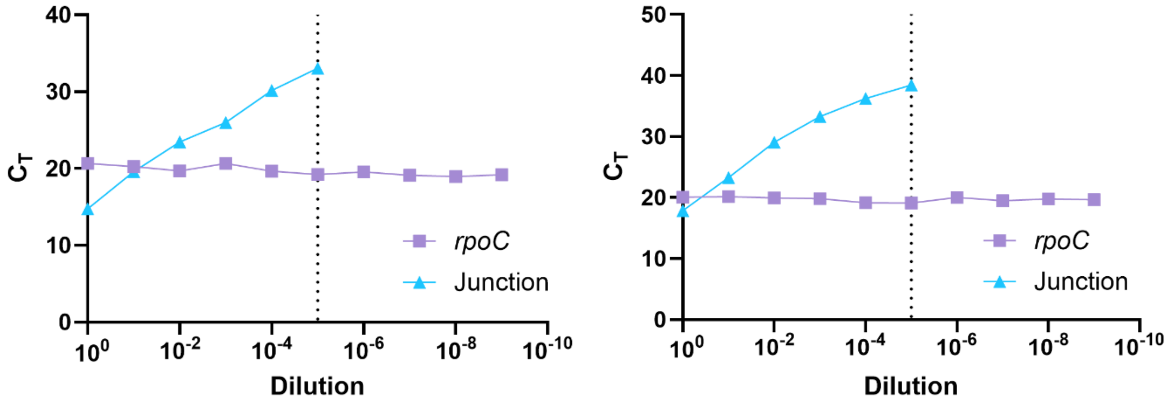

Supp. Fig. 6. Novel junctions corresponding to the *sdrM* amplifications can be detected at a 10^{-4} – 10^{-5} dilution. qPCR C_T values for *rpoC* and the respective novel junction present in two different WT evolved DLX resistant populations, measured in a 10-fold dilution series where genomic DNA from the evolved population was diluted in genomic DNA from the WT strain.

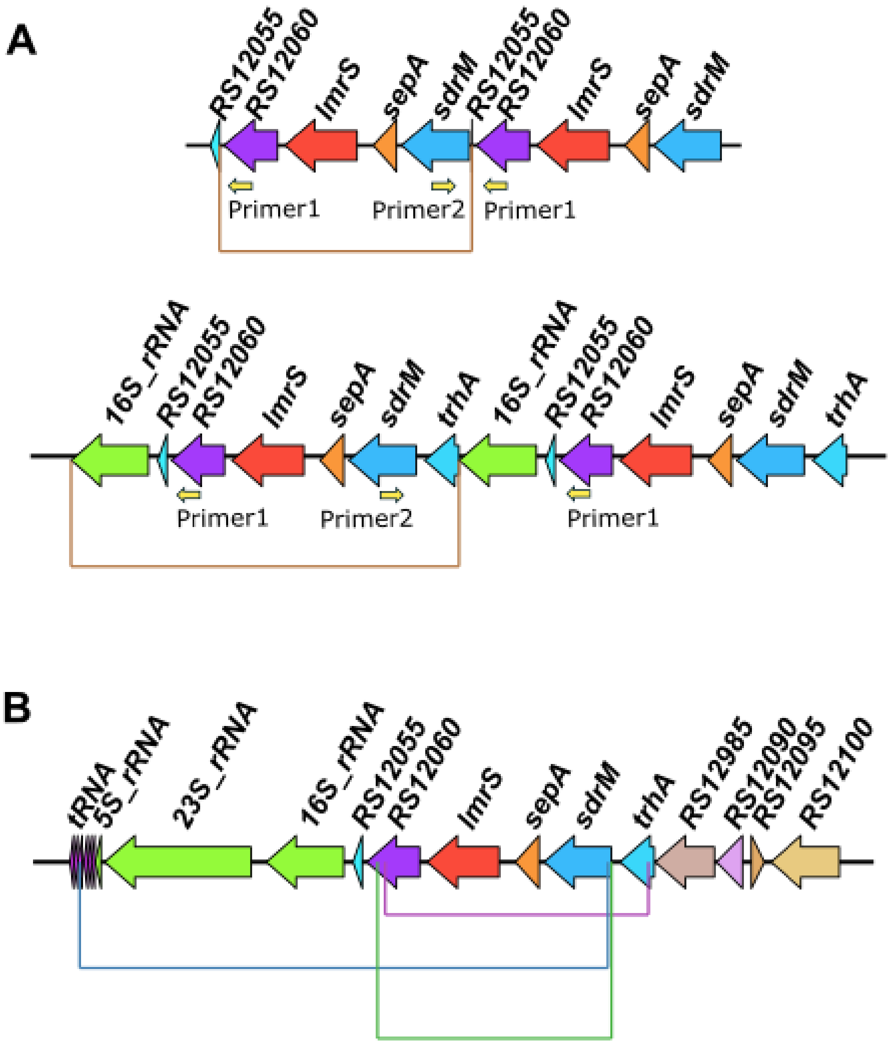

Supp. Fig. 7. Rare novel junctions around *sdrM* may be present in the unselected WT strain. **(A)** Inverse-PCR like strategy to detect novel junctions present around *sdrM*. Shown are two hypothetical amplifications (outlined by the brown lines). We designed outward facing primers (Primer 1 and Primer 2) binding to *sdrM* and *B7H15_RS12060*. If a duplication or amplification is present in that region, the novel junction should get amplified by the PCR primers. **(B)** Three putative duplications or amplifications identified in the WT strain using the inverse PCR strategy.

PHASTER New Search Genomes Help About My Searches

Download summary as .txt file: summary.txt

Total: 4 prophage regions have been identified, of which 2 regions are intact, 2 regions are incomplete, and 0 regions are questionable.

Region	Region Length	Completeness	Score	# Total Proteins	Region Position	Most Common Phage	GC %	Details
1	20.6Kb	incomplete	30	27	894188-914827	PHAGE_Staphy_PT1028_NC_007045(6)	30.80%	Show
2	7Kb	incomplete	50	18	1339497-1346518	PHAGE_Clostr_phiC2_NC_009231(1)	26.08%	Show
3	60.4Kb	intact	140	76	1558413-1618817	PHAGE_Staphy_phi2958PVL_NC_011344(31)	32.69%	Show
4	45.8Kb	intact	100	73	2086635-2132438	PHAGE_Staphy_phiNM3_NC_008617(42)	32.85%	Show

Intact (score > 90)
Questionable (score 70-90)
Incomplete (score < 70)

Supp. Fig. 8. The second region with increased coverage in several DLX resistant evolved populations encodes for a putative intact prophage. The JE2 genome was analyzed using PHASTER[4] to identify prophage regions. Region 4 (outlined in black) encodes an intact prophage whose coordinates match the region that shows increased sequencing coverage depth in the evolved DLX resistant populations.

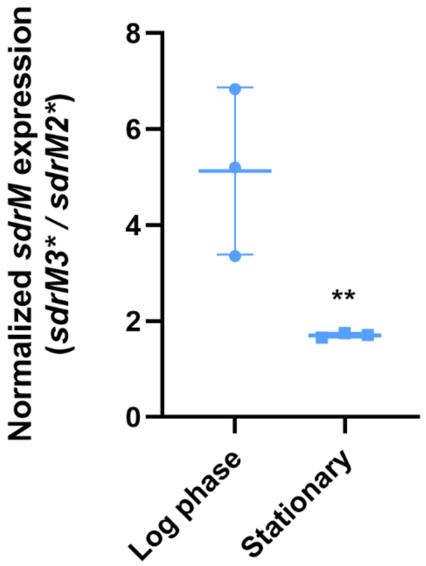

Supp. Fig. 9. An intergenic mutation upstream of *sdrM* leads to increased *sdrM* expression. Expression of *sdrM* was measured in log phase (OD_{600} = 0.5) and stationary phase (overnight cultures) of the previously reported *sdrM2** and *sdrM3** allele-replacement mutants[5]. The *sdrM2** mutant has the A268S coding sequence mutation, while the *sdrM3** mutant has the A268S mutation as well as a C to G change at position -164 (upstream of *sdrM*). Data shown are the means ± standard deviation for three biological replicates. Significance is shown for comparison to 1, as tested by a one-sample *t*-test (** $p < 0.01$).

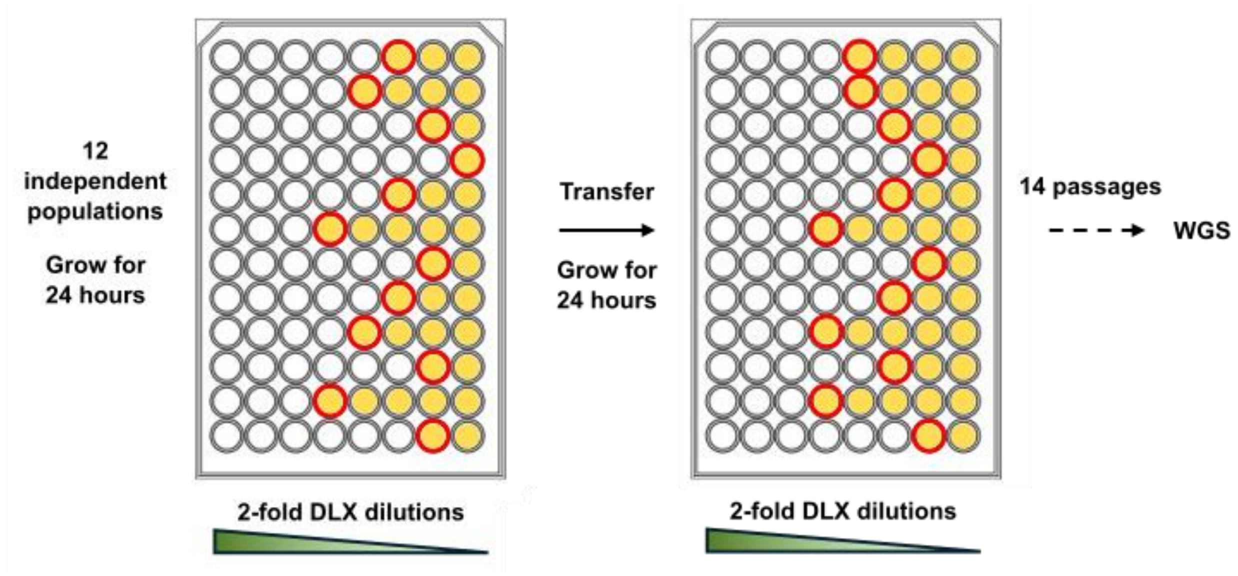

Supp. Fig. 10. Scheme for deep well evolutions. 12 independent populations of the strain to be evolved were inoculated in a 2-fold dilution series of DLX and grown for 24 hours. Populations from the well with the highest DLX concentration that showed growth were transferred to the next passage. Wells showing growth are denoted in yellow, and of those, the ones with the highest DLX concentration are marked with the red circles. Populations from these red-circled wells were propagated to the next passage. After 14 passages, the terminal passages from the wells with the highest DLX concentration that showed growth were sent for WGS.

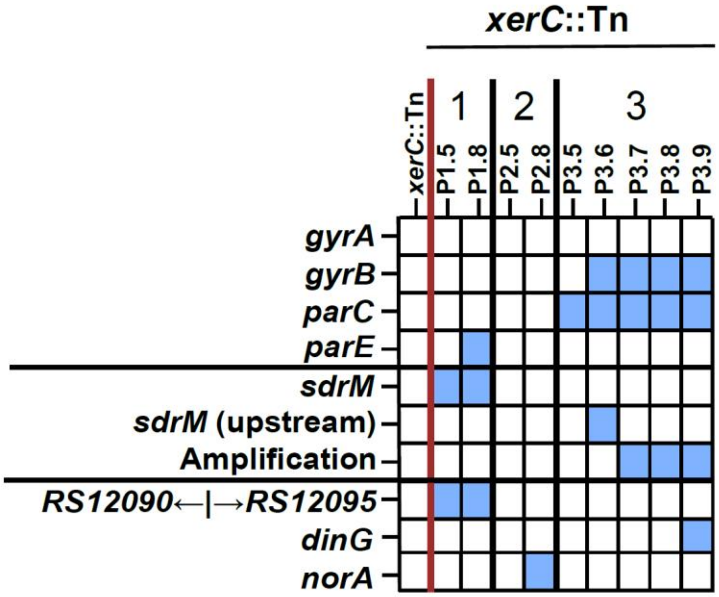

Supp. Fig. 11. The *xerC*::Tn mutant evolves DLX resistance via mutations in the canonical targets, *sdrM*, mutations increasing *sdrM* expression, and less prevalent *sdrM* amplifications. Common mutations seen in three independently evolved populations of the *xerC*::Tn mutant. Presence of mutations in the corresponding genes is indicated by a blue square. For each population, passages are shown from left to right in chronological order.

SUPPLEMENTARY TABLES

Supp. Table 1. Mutations seen in the *recA*::Tn evolved populations.

Supp. Table 2. Mutations seen in the evolved populations of R1 complemented with either an empty pKK30 plasmid or one carrying *recA*.

Supp. Table 3. List of strains evolved for DLX resistance.

Supp. Table 4. The distinct amplification fragments observed in the evolved populations from the tube evolutions of transposon and allele-replacement mutants.

Supp. Table 5. Mutations seen in the evolved populations from the tube evolutions of transposon and allele-replacement mutants.

Supp. Table 6. Mutations seen in the evolved populations of the allele-replacement strain with an intergenic mutation between *B7H15_RS12090* and *B7H15_RS12095*.

Supp. Table 7. Mutations seen in the evolved populations from the deep-well evolutions of transposon and complemented mutants.

Supplementary Table 8. Strains and plasmids used in this study.

Name	Description	Source
**Strains**		
S. aureus		
JE2	*Staphylococcus aureus* subsp. *aureus* USA300_FPR3757 (CA-MRSA)-JE2	
RN4220	Restriction modification deficient *S. aureus*; used to shuttle pKK30 derivatives into JE2	
SB514	1.7a (evolved DLX resistant mutant strain)	[5]
SB616	JE2 *lexA*^{S130A}	This study
SB617	JE2 *lexA*^{G94E}	This study
NE805	JE2 *recA*::Tn	[6]
NE331	JE2 *rexA*::Tn	[6]
NE1427	JE2 *recD2*::Tn	[6]
NE959	JE2 *sbcD*::Tn	[6]
NE324	JE2 *recX*::Tn	[6]
NE1673	JE2 *B7H15_RS12095*::Tn	[6]
NE883	JE2 *xerC*::Tn	[6]
NE445	JE2 *umuC*::Tn	[6]
NE1344	JE2 *recG*::Tn	[6]
NE346	JE2 *dinG*::Tn	[6]
NE972	JE2 *recQ*::Tn	[6]
NE1146	JE2 *B7H15_RS12090*::Tn	[6]
NE145	JE2 *uvrA*::Tn	[6]
NE1794	JE2 *ruvX*::Tn	[6]
NE555	JE2 *recF*::Tn	[6]
NE711	JE2 *B7H15_RS09290*::Tn	[6]
NE341	JE2 *rarA*::Tn	[6]
NE458	JE2 *xseA*::Tn	[6]
NE1679	JE2 *ssbB*::Tn	[6]
NE1830	JE2 *recT*::Tn	[6]
NE1528	JE2 *recQ2*::Tn	[6]
NE1866	JE2 *dinB*::Tn	[6]
NE188	JE2 *mfd*::Tn	[6]
NE97	JE2 *B7H15_RS12085*::Tn	[6]
NE1760	JE2 *B7H15_RS08985*::Tn	[6]
NE993	JE2 *trhA*::Tn	[6]
NE1689	JE2 *B7H15_RS10630*::Tn	[6]
NE1616	JE2 *sepA*::Tn	[6]
NE100	JE2 *B7H15_RS03470*::Tn	[6]
NE348	JE2 *B7H15_RS06660*::Tn	[6]
NE1529	JE2 *B7H15_RS12060*::Tn	[6]
NE1915	JE2 *B7H15_RS03710*::Tn	[6]
NE22	JE2 *polA*::Tn	[6]
NE152	JE2 *B7H15_RS12505*::Tn	[6]
SB618	Evolved *recA*::Tn isolate RecA1.P14a (R1)	This study

SB619	Evolved *recA*::Tn isolate RecA2.P13a (R2)	This study
SB620	Evolved *recA*::Tn isolate RecA3.P14a (R3)	This study
SB622	1.7a *recA*::Tn	This study
SB482	JE2 *recD2*^{A227E}	This study
SB483	JE2 *rexB*^{G576D}	This study
SB484	JE2 *B7H15_RS12090* ← / → *B7H15_RS12095*, at position 2,306,688, G→A	This study
SB486	*recA*::Tn *recD2*^{A227E}	This study
SB487	*recA*::Tn *rexB*^{G576D}	This study
SB489	*recA*::Tn B7H15_12090 ← / → B7H15_12095, at position 2,306,688, G→A	This study
SB621	Evolved DLX-resistant *xerC*::Tn mutant XC5_7	This study
E. coli		
DH5α-λpir	DH5α lysogenized with λpir; host for pKK30	[7]
IM08B	For plasmid transfer into *S. aureus*	[8]
**Plasmids**		
pIMAY*	*S. aureus* allelic exchange plasmid; Cm^R	[9]
pKK30	Expression vector for *S. aureus*; Tmp^R	[10]
pSB627	pIMAY*-*recD2*^{A227E}	This study
pSB626	pIMAY*-*rexB*^{G576D}	This study
pSB628	pIMAY*- *RS12090 ←/→ RS12095*	This study
pSB623	pIMAY*-*lexA*	This study
pSB624	pIMAY*-*lexA*^{S130A}	This study
pSB625	pIMAY*-*lexA*^{G94E}	This study
pSB469	pKK30-*recA*	This study
pSB598	pKK30-*xerC*	This study
pSB600	pKK30- *RS12060*	This study

Cm^R: chloramphenicol resistant; Tmp^R: trimethoprim resistant

Supplementary Table 9. Primers used in this study.

Primer Description	Primer Sequence*
Amplify *rexB* for pIMAY* F	TATCGATAAGCTTGATATCGAATTTAAACAT AATTTCAATCCTGAAAATAC
Amplify *rexB* for pIMAY* R	TGGAGCTCCACCGCGGTGGCCTCAACTAG TACAGCTGTTTTAC
Check pIMAY*-*rexB*^{G576D} integration F	ATACAACATCGTTTGTCGGTTT
Check pIMAY*-*rexB*^{G576D} integration R	TTGCGCGTCAGTCCAAAT
Sanger sequencing for *rexB*^{G576D}	GGCGTAAAGCGCATAAACTG
Amplify *recD2* for pIMAY* F	TATCGATAAGCTTGATATCGAAAAATTGATG ACACAGATAGTC
Amplify *recD2* for pIMAY* R	TGGAGCTCCACCGCGGTGGCAACTCAACT TAAACATGTATTAAC
Check pIMAY*-*recD2*^{A227E} integration F	CGGTACCACCCTAGTTATAAATGC
Check pIMAY*- *recD2* ^{A227E} integration R	ATGCAAGATACAATTTGGGCTTAG
Sanger sequencing for *recD2*^{A227E}	CTTGGAATTGCAACTTGTTCATTG
Amplify *RS12090 ←/→ RS12095* for pIMAY* F	TATCGATAAGCTTGATATCGCTATAAAAACT TAGTATTCCAGTTG
Amplify *RS12090 ←/→ RS12095* for pIMAY* R	TGGAGCTCCACCGCGGTGGCTTTAATTTCA CTTGTAGGAATTTTG
Check pIMAY*-*RS12090 ←/→ RS12095* integration F	TCTAATGACGCTACCTTCTCTTC
Check pIMAY*-*RS12090 ←/→ RS12095* integration R	TGTATTCGTGGTCTTCAGTATCG
Sanger sequencing for *RS12090 ←/→ RS12095*	TTCCGACCATTAACGCTGTAG
Mutagenesis of *lexA* to mutant allele S130A F	TAATATACCAGCCTCAATCATAGCGTCGCC TACGACGTTTAATATG
Mutagenesis of *lexA* to mutant allele S130A R	CATATTAAACGTCGTAGGCGACGCTATGAT TGAGGCTGGTATATTA
Mutagenesis of *lexA* to mutant allele G94E F	TTTCTACTGCGGTAATAGGAACCTCTGCTG TGACTTTACCAATAAC
Mutagenesis of *lexA* to mutant allele G94E R	GTTATTGGTAAAGTCACAGCAGAGGTTCCT ATTACCGCAGTAGAAA
Amplify *lexA* for pIMAY* F	TGGAGCTCCACCGCGGTGGCTTACATTTCG CGGTACAAAC
Amplify *lexA* for pIMAY* R	TATCGATAAGCTTGATATCGATGAGAGAATT AACAAAACGAC
Sanger sequencing for both *lexA* mutant alleles	TTTCGCGGTACAAACCAATTAC
Check pIMAY*-*lexA* integration_F	ATAATGGCTCGCTCCTGTAAAT
Check pIMAY*-*lexA* integration_R	TCCCATCTCTTTGTTCGCTATT
Check transposon insertion for *recF*::Tn _F	TGAAACACGTCGCGGTAAA
Check transposon insertion for *recF*::Tn _R	CATCTCGATGTGGTCCGAATAA
Check transposon insertion for *xerC*::Tn _F	TTCTTAAGTCATCAACGAGTAA
Check transposon insertion for *xerC*::Tn _R	AAGCGTTACTTGCCCATCTC
Check transposon insertion for *recG*::Tn _F	CGCGTTTATGGGCTTGATTG
Check transposon insertion for *recG*::Tn _R	GTATCGGCGTTGCTGTCATA
Check transposon insertion for *sbcD*::Tn _F	GGAGAATTAGGCGGCATGTT

Check transposon insertion for *sbcD*::Tn _R	ACCTCTCTTTACCATCGTGATTT
Check transposon insertion for *recJ*::Tn _F	CCATCCTACTTGTGCACCTAA
Check transposon insertion for *recJ*::Tn _R	CTTCAATCTTCATTGCCGTT
Check transposon insertion for *recQ*::Tn _F	TCGTAATGTGCTTGGTGTCTTA
Check transposon insertion for *recQ*::Tn _R	TGGTAACTCGGCCTGAAATC
Check transposon insertion for *recX*::Tn _F	GTTGCGTGTACTTCTTTCGATTT
Check transposon insertion for *recX*::Tn _R	TGATCCAGTGCCGAAGATTAC
Check transposon insertion for *uvrA*::Tn _F	TGGGTCAGGTAAATCGTCATTAG
Check transposon insertion for *uvrA*::Tn _R	AAATCAGAACTCGTCGGTATCC
Check transposon insertion for *dinG*::Tn _F	GGCGTCTCTGTAATCCAAATCA
Check transposon insertion for *dinG*::Tn _R	GATGCCGCTACTACTGCTAAA
Check transposon insertion for *RS09290*::Tn _F	TGTAAGTGAGGAGGGATGTACT
Check transposon insertion for *RS09290*::Tn _R	CAATCGCTTGCTTCGTTCTTC
Check transposon insertion for *rarA*::Tn _F	ACCAAGAGGCATTATCAGAAGAA
Check transposon insertion for *rarA*::Tn _R	AGTTGTAGCACCGATCAAGAC
Check transposon insertion for *xseA*::Tn _F	GCACCTGTACTCGCTGTTAAA
Check transposon insertion for *xseA*::Tn _R	CTGGCGTTAAAGGTCACGAA
Check transposon insertion for *ssbB*::Tn _F	CAAAGTGTGTTTGTCCGTCTTTAT
Check transposon insertion for *ssbB*::Tn _R	AATCGTAATTGTCGGGAGACTG
Check transposon insertion for *recT*::Tn _F	CTTGCTTGAATGTATCGCCTTTAT
Check transposon insertion for *recT*::Tn _R	TTCACCAAGTAATGCCATGAAAC
Check transposon insertion for *recQ2*::Tn _F	AAGCACTCGTTGCGACTATAA
Check transposon insertion for *recQ2*::Tn _R	AGCGGTTGTCTTAGCATTGA
Check transposon insertion for *RS12085*::Tn _F	CCATTACCATTCGGCGTTTC
Check transposon insertion for *RS12085*::Tn _R	TGAAGAGAAGGTAGCGTCATTAG
Check transposon insertion for *dinB*::Tn _F	CTTCATCATCATTCACGTCTGTTG
Check transposon insertion for *dinB*::Tn _R	AGCATCTGCAGGTGTTTCTTA
Check transposon insertion for *mfd*::Tn _F	CTGCGTTAGCTCAAGGTAAGA
Check transposon insertion for *mfd*::Tn _R	CGCTGCGTTTCAACATCAA
Check transposon insertion for *ruvX*::Tn _F	AGCACCTTCTTCAGCTAAGATAAC
Check transposon insertion for *ruvX*::Tn _R	GGCACAAGGATTAGACACACTC
Check transposon insertion for *RS08985*::Tn _F	AGCGCCAATAACATCTTCTACT
Check transposon insertion for *RS08985*::Tn _R	AGTGATCCGACATTGTCCATTTA
Check transposon insertion for *RS06660*::Tn _F	TTTGGTGGCGGTGTATTAGG
Check transposon insertion for *RS06660*::Tn _R	TGCAGAACGACCAGGTATTG
Check transposon insertion for *trhA*::Tn _F	CAACCTATCCAACCACCTACAA
Check transposon insertion for *trhA*::Tn _R	AATGCGGCATCTCATGGT
Check transposon insertion for *RS12060*::Tn _F	TCCAGGTGGAAGATCGAGTAT
Check transposon insertion for *RS12060*::Tn _R	GGTAAAGGTGGTGTCGGTAAA
Check transposon insertion for *RS03470*::Tn _F	CCAGCTTGACGACCTTTCATA
Check transposon insertion for *RS03470*::Tn _R	GGCGTACGTATACAGCCTTATC
Check transposon insertion for *sepA*::Tn _F	ACGTTGTTGCAACTGTGTAAG
Check transposon insertion for *sepA*::Tn _R	TTTGTACTTTCTGGTGCGATTT
Check transposon insertion for *RS10630*::Tn _F	TCTCTGTCGCTTGTTGTTCTG
Check transposon insertion for *RS10630*::Tn _R	AGGGTATGTCGTGTGCAATG
Check transposon insertion for *RS03710*::Tn _F	TGCAGAAATGAAACTTGCTGTC
Check transposon insertion for *RS03710*::Tn _R	CTACAGCTATAGTGACGATGATT
Check transposon insertion for *RS12505*::Tn _F	CTTCTCGTCCAGCATCTGTT

Check transposon insertion for *RS12505*::Tn _R	GTGATTCAATCGCATCGGTTATT
Check transposon insertion for *polA*::Tn_F	TCTGGCGTCCACCTTTATATTC
Check transposon insertion for *polA*::Tn_R	TGGAGTGCTCATCAAGAACAA
Check transposon insertion for *recA*::Tn_F	TCGGTAAAGGTGCCGTAATG
Check transposon insertion for *recA*::Tn_F	ACGTAACGCTTGTGACATTAAAC
Check transposon insertion for *rexA*::Tn_F	GGTGACACCATACGAAGAAGAA
Check transposon insertion for *rexA*::Tn_F	CTCTTGAACTCGGTTCGTATCT
Check transposon insertion for *RS12090*::Tn _F	TTCCGACCATTAACGCTGTAG
Check transposon insertion for *RS12090*::Tn _R	ACTCTCCATTGAATACGCACTT
Check transposon insertion for *RS12095*::Tn _F	TTCCGACCATTAACGCTGTAG
Check transposon insertion for *RS12095*::Tn _R	TGTATTCGTGGTCTTCAGTATCG
Check transposon insertion for *recD2*::Tn_F	CGGTACCACCCTAGTTATAAATGC
Check transposon insertion for *recD2*::Tn_F	ATGCAAGATACAATTTGGGCTTAG
Check transposon insertion for *umuC*::Tn_F	GTTGTTGCAGATACTAAGCG
Check transposon insertion for *umuC*::Tn_R	AAAGCATTGAACACCATAACCG
Gibson assembly for pKK30 F	GCGGCCGCTAGCCTAGGAGC
Gibson assembly for pKK30 R	ATCGCCTGTCACTTTGCTTGATATATGA
Amplify *xerC* for pKK30 F	CAAGCAAAGTGACAGGCGATAAACTAGAATTAAAGATAAAAAAGAACG
Amplify *xerC* for pKK30 R	GCTCCTAGGCTAGCGGCCGCTCATGTTTCATTCTCCTTTTTC
Amplify RS12060 for pKK30 F	ATCAAGCAAAGTGACAGGCGATCTATTTATTTGTAGCAGCTATAAC
Amplify RS12060 for pKK30 R	GAGCTCCTAGGCTAGCGGCCGCTCTCATTTGCTTTTGTAGC
Amplify *recA* for pKK30 F	CAAGCAAAGTGACAGGCGATTTGTATTATCGATAAAAATATAAGCAC
Amplify *recA* for pKK30 R	GCTCCTAGGCTAGCGGCCGCCTATTCTTCGTCAAATAATGAC
Check junctions in JE2 WT_F	AATTCGCCCACTATAGGATTGG
Check junctions in JE2 WT_R1	GGAGGCCAATGATTGTGAATTA
Check junctions in JE2 WT_R2	TTTGAAACATTGCCGGAAGAAA
qPCR for junction in DLX resistant population WT2_F	GCAGTTGCAACACTATCTTTACC
qPCR for junction in DLX resistant population WT2_R	CGATGAGTGCTAAGTGTTAGGG
qPCR for junction in DLX resistant population WT3_F	TACTCCAGTACTGTATCTTTACAATCG
qPCR for junction in DLX resistant population WT3_R	AACTGAATGACAATATGTCAACG
qPCR for housekeeping gene *rpoC*_F	CTGTGAAAGAATTTTCGGAC
qPCR for housekeeping gene *rpoC*_R	CTTTCACGACGTACTTTAGA
qPCR for *sdrM*_F	GCAATGATCGCAATCGGTAT
qPCR for *sdrM*_R	GGCATAGTTGGCAGTGTTTG
qPCR for *trhA*_F	AGACATACTCACAGACGCAAG
qPCR for *trhA*_R	AATGCGGCATCTCATGGT
qPCR for *RS12085*_F	TTCTTCTAAGTATCCTGCCTTGTC
qPCR for *RS12085*_R	ATTGTGGCGCTTAGTGAAGA
qPCR for *RS12090*_F	AGGTTCGATGTCCAAAGATCAA

qPCR for *RS12090*_R	GCAACTCTAGCTAGCTTATTACCC
qPCR for *RS12095*_F	GCAGTTCCTAAATTAGACAGCAAAG
qPCR for *RS12095*_R	ATGTATTCGTGGTCTTCAGTATCG

*Underlined sequences represent homology for Gibson assembly.

REFERENCES

1 Marchler-Bauer, A. & Bryant, S. H. CD-Search: protein domain annotations on the fly. *Nucleic Acids Research* **32**, W327-W331 (2004).

2 Wang, J. *et al.* The conserved domain database in 2023. *Nucleic Acids Research* **51**, D384-D388 (2023).

3 Sprouffske, K. & Wagner, A. Growthcurver: an R package for obtaining interpretable metrics from microbial growth curves. *BMC Bioinformatics* **17**, 172 (2016). https://doi.org:10.1186/s12859-016-1016-7

4 Arndt, D. *et al.* PHASTER: a better, faster version of the PHAST phage search tool. *Nucleic Acids Research* **44**, W16-W21 (2016).

5 Silva, K. P. T., Sundar, G. & Khare, A. Efflux pump gene amplifications bypass necessity of multiple target mutations for resistance against dual-targeting antibiotic. *Nature Communications* **14**, 3402 (2023). https://doi.org:10.1038/s41467-023-38507-4

6 Fey, P. D. *et al.* A genetic resource for rapid and comprehensive phenotype screening of nonessential *Staphylococcus aureus* genes. *mBio* **4**, e00537-00512 (2013).

7 Dunn, A. K., Martin, M. O. & Stabb, E. V. Characterization of pES213, a small mobilizable plasmid from *Vibrio fischeri*. *Plasmid* **54**, 114-134 (2005).

8 Monk, I. R., Tree, J. J., Howden, B. P., Stinear, T. P. & Foster, T. J. Complete bypass of restriction systems for major *Staphylococcus aureus* lineages. *mBio* **6**, e00308-00315 (2015).

9 Schuster, C. F., Howard, S. A. & Gründling, A. Use of the counter selectable marker PheS* for genome engineering in *Staphylococcus aureus*. *Microbiology* **165**, 572-584 (2019).

10 Krute, C. N. *et al.* Generation of a stable plasmid for in vitro and in vivo studies of *Staphylococcus* species. *Applied and Environmental Microbiology* **82**, 6859-6869 (2016).
